# Biodiversité de la flore fongique isolée au service de réanimation du Centre Hospitalo-Universitaire Souro Sanou de Bobo-Dioulasso, Burkina Faso

**DOI:** 10.11604/pamj.2021.38.299.27596

**Published:** 2021-03-23

**Authors:** Hadry Roger Sibi Matotou, Ibrahim Sangare, Cyrille Bisseye, Marielle Karine Bouyou Akotet, Sanata Bamba

**Affiliations:** 1 Institut Supérieur des Sciences de la Santé, Université Nazi Boni, 01 BP 1091, Bobo-Dioulasso, Burkina Faso,; 2 Université des Sciences de la Santé, Département de Parasitologie-Mycologie, BP 4009 Libreville, Gabon,; 3 Service de Parasitologie-Mycologie, Département des Laboratoires, Centre Hospitalier Universitaire Souro Sanou, 01 BP 676, Bobo-Dioulasso, Burkina Faso,; 4 Laboratoire de Biologie Moléculaire et Cellulaire, Université des Sciences et Techniques de Masuku, BP 943, Franceville, Gabon

**Keywords:** Biodiversité, champignons, réanimation, Biodiversity, fungi, resuscitation

## Abstract

**Introduction:**

les maladies nosocomiales demeurent un problème majeur de santé publique en Afrique subsaharienne notamment au Burkina Faso. Cette étude avait pour but de déterminer la biodiversité de la flore fongique identifiée au service de réanimation du Centre Hospitalier Universitaire Souro Sanou (CHUSS) de Bobo-Dioulasso.

**Méthodes:**

l´étude transversale descriptive s´est déroulée d´août 2016 à janvier 2017. La flore fongique a été recherchée dans l´air ambiant et les espaces de réanimation. Les prélèvements ont été ensemencés et incubés pendant 3 à 4 jours à 37°C à l´étuve. L´identification des colonies fongiques était macroscopique et microscopique pour les champignons filamenteux. Le test de blastèse, les milieux chromogéniques et le test d´agglutination au latex ont servi à l´identification d´espèces de Candida.

**Résultats:**

sur les 200 prélèvements collectés au total, 176 ont poussé sur la gélose Sabouraud-Chloramphénicol. La prévalence globale de la flore fongique était de 88% (176/200). Les moisissures étaient les agents fongiques majoritairement retrouvés (66,9%). Parmi les huit genres de moisissures identifiés, Aspergillus était le genre le plus représenté (48,9%) tandis qu´Aspergillus fumigatus était l´espèce la plus fréquemment rencontrée (32,9%).

**Conclusion:**

la décontamination régulière des niches fongiques devraient être systématiques dans le service de réanimation du CHUSS de Bobo-Dioulasso.

## Abstract (en)

**Introduction:**

nosocomial diseases are a major public health problem in sub-Saharan Africa particularly in Burkina Faso. The purpose of this study was to determine the biodiversity of the fungal flora identified at the reanimation service of the University Hospital Souro Sanou of Bobo-Dioulasso.

**Methods:**

we conducted a descriptive cross-sectional study from August 2016 to January 2017. The purpose of the study was to investigate the fungal flora in the ambient air and in the reanimation environment. Samples were seeded and incubated for 3-4 days at 37°C in the oven. The identification of colonies of filamentous fungi was macroscopic and microscopic. Blastesis test, chromogenic medium and latex agglutination test were used to identify Candida species.

**Results:**

out of 200 samples collected, 176 grew in Sabouraud-Chloramphenicol agar. The overall prevalence of fungal flora was 88% (176/200). Molds were the most common fungal agents found (66.9%). Among the eight types of mold identified, Aspergillus was the most common family (48.9%) while Aspergillus fumigatus was the most detected species (32.9%).

**Conclusion:**

regular decontamination of fungal niches should be systematic at the reanimation service of the University Hospital Souro Sanou of Bobo-Dioulasso.

## Introduction

Les infections nosocomiales sont des infections liées aux soins et à l´air ambiant hospitalier; elles demeurent des maladies redoutables [1, 2]. En réanimation, la maitrise de la qualité de l´air ambiant dans les salles d´hospitalisation est primordiale. En effet, les champignons aéroportés constituent un danger réel pour les patients hébergés dans ces services notamment les immunodéprimés [3, 4]. Ces agents fongiques représentent à l´heure actuelle une préoccupation majeure pour les services de soins intensifs et un défi important pour les cliniciens et les microbiologistes. Le caractère émergent de ces agents infectieux, associé à leur abondance et à leur résistance variable à certains antifongiques (ATF) notamment les azolés, constitue l´une des causes d´échecs thérapeutiques rendant délicate la prise en charge des patients admis dans les services de réanimation [5, 6].

Par ailleurs, les comportements du personnel, la mauvaise hygiène du matériel et dans les locaux, tout comme l´insuffisance de formations sur les bonnes pratiques en milieux de soins pourraient être à l´origine de l´émergence de nombreux agents fongiques enregistrés en réanimation [3]. Cependant, peu d´études ont été faites au Burkina sur les micromycètes de l´environnement hospitalier notamment dans les services de réanimation, alors qu´une une étude antérieure a montré une grande diversité fongique au niveau des sites corporels superficiels [7]. L´objectif de cette étude était de déterminer la biodiversité de la flore fongique au service de réanimation polyvalente (SRP) du Centre Hospitalier Universitaire Souro Sanou (CHUSS) de Bobo-Dioulasso.

## Méthodes

**Population d´étude:** il s´est agi d´une étude transversale à visée descriptive qui s´est déroulée du 1^er^ août 2016 au 31 janvier 2017 au SRP et au laboratoire de Parasitologie-Mycologie du CHUSS de Bobo-Dioulasso. Les prélèvements ont concerné l´air ambiant; le poignet de la porte principale d´entrée du service; le tuyau du climatiseur et la paillasse du personnel soignant. Une fiche de collecte a été établie pour le recueil des données. La collecte des échantillons a été réalisée par écouvillonnage (surfaces) et exposition en boite de Pétri contenant de la gélose Sabouraud + Chloramphénicol.

**Analyse mycologique:** les prélèvements collectés ont été ensemencés et incubés au laboratoire dans une étuve à 37°C pendant 3 à 4 jours pour les levures et les moisissures et jusqu´à trois semaines pour les dermatophytes. Les champignons isolés après culture ont été identifiés de façon macroscopique grâce à l´aspect de leurs colonies. Ainsi, en présence des champignons levuriformes, on observait des colonies blanchâtres, lisses, glabres, humides, d´aspects brillants ou mats et parfois rugueux. Tandis qu´en présence des champignons filamenteux, des colonies de texture duveteuse, laineuse, cotonneuse ou granuleuse de couleurs variables ont été observées selon les espèces. Les champignons filamenteux isolés après culture ont été identifiés grâce à leur apparence microscopique dans du bleu de lactophénol après scotch-test sur une lame porte objet au microscope à l´objectif 40. Ainsi en présence des champignons du genre *Aspergillus*, l´identification était basée sur la mise en évidence des conidiophores, de longueur variable selon les espèces, qui se terminaient par des têtes aspergillaires (les têtes unisériées ou bisériées) constituées d´un ensemble de vésicule, de phialides et de conidies. L´identification rapide du complexe *Candida albicans-Candida dubliensis* a reposé sur le fait que *Candida albicans* incubé 2 à 4 h à 37°C dans 2 ml de sérum humain produit des tubes germinatifs sans constriction à la base [7].

**Analyse statistique:** les données ont été traitées et analysées avec les logiciels IBM SPSS 20, EPI info Tm 7.

## Résultats

**Genres et espèces fongiques isolés au service de réanimation:** sur les deux cent (200) échantillons collectés au service de réanimation (air et espace), 176 étaient positifs (88%) dont 82,4% (145/200) en monoculture. Sur les 145 monocultures positives, les groupes fongiques les plus fréquents étaient respectivement les moisissures (66,9%), les levures (24,8%) et les dermatophytes/ pseudodermatophytes (8,3%) (Tableau 1). Parmi les moisissures, *Aspergillus* étaient le genre fongique le plus représenté (48,9%) tandis *Candida* et *Scytalium* étaient les genres les plus fréquents chez les levures et les dermatophytes/ pseudodermatophytes avec respectivement 24,1% et 6,2% (Tableau 1). Dans le genre *Aspergillus*, les espèces les plus fréquentes étaient *Aspergillus fumigatus* et *Aspergillus flavus* avec respectivement 32,9% et 4,4% tandis que *Fusarium oxysporum, Scedosporium apiospermum, Penicillium sp, Rhizomucor miehei* étaient les espèces les moins fréquentes avec respectivement 1,1% chacune (Tableau 2). En ce qui concerne les levures, l´espèce *Candida albicans* (30,6%) était la plus représentée, et les espèces les moins observées étaient respectivement *Candida krusei, Candida glabrata, Geotricum candidum* avec 2,8% chacune (Tableau 2). Les agents fongiques ont été aussi isolés en culture mixte et les principales espèces retrouvées ont été les associations *Aspergillus terreus/Aspergillus niger* (35,5%); *Aspergillus terreus/Aspergillus* flavus (29,1%) et *Aspergillus fumigatus/Aspergillus terreus* (25,8%) (Tableau 3).

**Tableau 1:** répartition des principaux genres fongiques isolés en monoculture

Genres fongiques	Nombre	Pourcentage
**Levures**	36	24,8
*Candida*	35	24,1
*Geotricum*	1	0,7
**Moisissures**	97	66,9
*Aspergillus*	71	48,9
*Rhizopus*	11	7,6
*Mucor*	9	6,2
*Absidia*	2	1,4
*Fusarium*	1	0,7
*Penicillium*	1	0,7
*Rhizomucor*	1	0,7
*Scdeosporium*	1	0,7
**Dermatophytes et pseudodermatophytes**	12	8,3
*Scytalium*	9	6,2
*Trichophyton*	3	2,1

**Tableau 2:** distribution des différentes espèces de moisissures et de levures identifiées en monoculture

Espèces fongiques	Nombre	Pourcentage
**Moisissures**	**N = 97**	
*Aspergillus fumigatus*	32	32,9
*Aspergillus flavus*	14	14,4
*Aspergillus niger*	13	13,4
*Aspergillus terreus*	12	12,4
*Rhizopus sp*	11	11,3
*Mucor sp*	9	9,2
*Absidia corymbifera*	2	2
*Rhizomucor miehei*	1	1,1
*Fusarium oxysporum*	1	1,1
*Scedosprorium apiospermum*	1	1,1
*Penicillium sp*	1	1,1
**Levures**	**N = 36**	
*Candida spp*	20	55,5
*Candida albicans*	11	30,6
*Candida dubliniensis*	2	5,5
*Candida krusei*	1	2,8
*Candida glabrata*	1	2,8
*Geotricum candidum*	1	2,8

**Tableau 3:** distribution des cultures mixtes pour l’ensemble des secteurs prélevés

Cultures mixtes	Nombres	Pourcentage
*Aspergillus terreus/Aspergillus niger*	11	35,5
*Aspergillus terreus/Aspergillus flavus*	9	29,1
*Aspergillus fumigatus/Aspergillus terreus*	8	25,8
*Aspergillus. flavus/Aspergillus fumigatus*	1	3,2
*Aspergillus fumigatus/Aspergillus niger*	1	3,2
*Aspergillus terreus/Rhizopus sp*	1	3,2

**Répartition des cultures positives en fonction des sites de prélèvements:** les cultures positives provenaient majoritairement de l´air ambiant (51%) du service de réanimation. Les monocultures positives ont été retrouvées dans l´air ambiant (49%); sur la palliasse du personnel soignant (19,3%); sur le poignet de la porte principale d´entrée (15,2%) et au niveau du tuyau de la climatisation (16,6%).

**Répartition des genres fongiques en fonction des sites de prélèvements:** la répartition des groupes fongiques a été déterminée en fonction des sites de prélèvements. Les moisissures ont été retrouvées sur tous les sites. Elles étaient les plus fréquentes dans l´air ambiant (84,5%), les tuyaux des climatiseurs (79,2%) et les poignets des portes (68,2%) (Tableau 4). Tout comme les moisissures, les levures ont été isolées sur tous les sites de prélèvements. Elles étaient majoritairement retrouvées sur la paillasse du personnel (85,7%) et marginalement dans l´air ambiant (5,6%). Les dermatophytes/pseudodermatophytes était le groupe fongique le moins isolé et a été retrouvé plus fréquemment sur les poignets des portes (18,2%) (Tableau 4). En ce qui concerne les principales espèces fongiques isolées au service de réanimation, *A. fumigatus* (22,1%) a été plus retrouvé dans l´air ambiant, *Candida spp*. (13,8%) sur la paillasse du personnel et *Scytalidium hyalinum* (6,2%) sur les poignets des portes.

**Tableau 4:** répartition des genres de monocultures positives selon la provenance des prélèvements

Provenance des prélèvements	Monocultures positives	Moisissures N (%)	Levures N (%)	Dermatophytes/ pseudodermatophyte N (%)
Air ambiant	71	60 (84,5)	4 (5,6)	7 (9,7)
Paillasse du personnel	28	3 (10,7)	24 (85,7)	1 (3,6%)
Poignets des portes	22	15 (68,2)	3 (13,6)	4 (18,2%)
Tuyau des climatiseurs	24	19 (79,2)	5 (20,8)	0 (0,0)
Total	145	97	36	12

## Discussion

Dans cette étude nous avons trouvé que la prévalence globale des agents fongiques au CHUSS de Bobo-Dioulasso au Burkina Faso était de 88%. Cette prévalence était largement supérieure à celle observée au CHU de Bouaké en Côte d´Ivoire qui était de 48,4% [4]. La plus forte prévalence d´agents fongiques trouvée au Burkina Faso comparativement à celle rapportée en Côte d´Ivoire pourrait s´expliquer en partie par la différence entre les saisons d´études (hivernale et sèche au Burkina Faso versus saison sèche en Côte d´Ivoire). En effet, les saisons hivernales (humides) et sèches (Harmattan) au Burkina Faso sont favorables à la prolifération et à la dispersion des spores; tandis que la saison sèche en Côte d´Ivoire pourrait être peu favorable à la survie et à la dispersion des spores dans l´environnement. En ce qui concerne les groupes fongiques identifiés, les moisissures étaient les plus fréquentes (66,9%), suivies des levures (24,8%) et des pseudo dermatophytes/dermatophytes (8,3%).

La prédominance des moisissures dans cette étude a été aussi rapportée par les études antérieures [3, 4, 8, 9]. En effet, lorsque les conditions climatiques sont favorables, les moisissures produisent, à maturité, de très nombreuses spores très légères qui peuvent être transportées par les courants d´air ou par les humains jusqu´aux habitations et édifices tels que les hôpitaux [8, 9]. Les moisissures pourraient aussi avoir comme source des dispositifs médicaux générateurs d´aérosols qui constitueraient leur support de dissémination [10]. Dans la présente étude, *Aspergillus* était le genre de moisissures le plus fréquemment isolé (48,9%), suivi de *Rhizopus* (7,60%), de *Mucor* (6,20%) et d´*Absidia* (1,40%). La prédominance du genre *Aspergillu*s a également été observée dans les études antérieures faites en Grèce [11]. Au Brésil, le genre *Penicillium* était le plus retrouvé [12-14] alors qu´au Sénégal c´est le genre *Clasdosporium* qui avait été majoritairement isolé [3]. En Turquie, dans une étude faites en milieu hospitalier, les genres *Aspergillus, Penicillium, Alternaria, Cladosporium* étaient les moisissures les plus fréquemment identifiées [15].

D´autres études antérieures ont montré qu´une trentaine de genres fongiques étaient régulièrement observés en milieu fermé dont les plus fréquents étaient *Cladosporium, Alternaria, Curvularia, Aspergillus* et *Penicillium* [16, 17]. Cela pourrait être dû au fait qu´*Aspergillus, Penicillium* et *Cladosporium* produisent de nombreuses petites spores légères qui demeurent dans l'air pendant longtemps, tandis que les autres genres de champignons produisent une faible quantité de grandes spores lourdes ayant une dispersion plus rapide [18]. Dans cette étude, les autres genres de moisissures tels que *Rhizomucor, Fusarium, Penicillium* et *Scedosporium* ont été peu retrouvés ceci est en accord avec les résultats d´une étude antérieure faite en Iran [19]. Les prévalences des levures et des dermatophytes/pseudodermato phytes étaient respectivement de 24,80% et 8,3%. La fréquence déterminée en levures dans la présente étude était supérieure à celle de 11,1% retrouvée au Sénégal [3]; mais inférieure à la fréquence de 36,4% observée en milieu hospitalier en Côte d´Ivoire [4]. Dans une étude faite en Iran, ni les levures, ni les pseudodermatophytes n´avaient été isolés. L´absence de ces agents fongiques s´expliquerait par le fait que les prélèvements concernaient uniquement l´air où ces agents fongiques sont peu présents [19].

Parmi les levures, l´espèce *Candida spp*. (55,5%) était prédominante suivie de l´espèce *Candida albicans* (30,6%). Cette prédominance de *Candida spp*. serait liée à la limite des techniques diagnostiques utilisées dans cette étude. En effet, les galeries d´identification fongique API et la spectrométrie de masse type MALDI TOF [20] auraient permis une identification plus précise de *Candida spp*. Parmi les levures, *Geotricum candidum* était minoritaire avec une prévalence de 2,80%. Cette levure était prédominante (36,4%) par rapport aux *Candida* dans une étude antérieure faite en Côte d´Ivoire [4]. La présence des levures dans l´environnement hospitalier pourrait être associée aux soins (fibroscopie et oxygénothérapie); à l´usage des dispositifs médicaux (sonde); aux patients et/ou au personnel soignant [21]. *Scytalidium hyalinum* a été le pseudodermatophyte détecté dans cette étude, il s´agit de moisissures kératinophiles dont l'habitat naturel n'est pas encore déterminé de façon précise. Leurs spores pourraient se retrouver en milieu hospitalier à travers un réservoir humain contaminé.

## Conclusion

Dans cette étude, une forte présence d´agents fongiques disséminés dans la flore environnementale du service de réanimation du CHUSS a été observée. Les moisissures étaient le groupe fongique le plus isolé avec la prédominance du genre *Aspergillus*. L´air a constitué le site le plus contaminé. Au regard des résultats obtenus, des mesures de lutte contre la dissémination des champignons dans l´environnement hospitalier sont indispensables. Ces mesures consisteraient au renouvellement de l´air, à la désinfection des paillasses de travail mais également à la sensibilisation du personnel sur les mesures d´hygiène et comportementale telles que les lavages de mains et la réduction de la mobilité entre les salles.

### Etat des connaissances sur le sujet

- Aucune étude sur les micromycètes n´a été réalisée dans les services de réanimation au Burkina Faso;
- Les micromycètes sont présents dans l´environnement de l´homme à des proportions distinctes;
- Les levures du genre Candida sont présentes sur des surfaces humides favorables à leurs croissances.

### Contribution de notre étude à la connaissance

- Notre étude a rapportée pour la première fois la prévalence des agents fongiques dans un service de réanimation au Burkina Faso;
- Les moisissures et Aspergillus fumigatus ont été respectivement le groupe et l´espèce fongique les plus isolés;
- Notre travail confirme la prépondérance des espèces Candida albicans et Aspergillus fumigatus dans l´air ambiant et les paillasses des services hospitaliers.

## Remerciements

Les auteurs remercient tout le staff du service de réanimation du CHUSS pour leur précieuse aide pendant la réalisation de ce travail.
